# Leveraging Whole-Exome Sequencing to Decipher the Genetic Landscape of Three Genodermatoses’ Cases in Middle Eastern Pediatric Patients

**DOI:** 10.3390/genes17050535

**Published:** 2026-04-30

**Authors:** Ayat Kadhi, Pierre Abi Akl, Ossama Abbas, Elias El-Tayar, Georges Nemer, Mazen Kurban

**Affiliations:** 1 College of Health and Life Sciences, Hamad Bin Khalifa University, Doha P.O. Box 34110, Qatar; ayat.kadhi@udst.edu.qa; 2 Department of Human Genetics, Sidra Medicine, Doha P.O. Box 26999, Qatar; 3 College of Health and Sciences, University of Doha for Science and Technology, Doha P.O. Box 24449, Qatar; 4 Department of Otorhinolaryngology—Head and Neck Surgery, American University of Beirut, Beirut P.O. Box 11-0236, Lebanon; pa39@aub.edu.lb; 5 Department of Dermatology, American University of Beirut, Beirut P.O. Box 11-0236, Lebanon; oa09@aub.edu.lb (O.A.); ee42@aub.edu.lb (E.E.-T.)

**Keywords:** keratitis–ichthyosis–deafness (KID) syndrome, epidermolysis bullosa, whole-exome sequencing (WES)

## Abstract

**Introduction:** Recent advancements in genomic technologies have significantly improved the resolution of genetic variants driving rare genodermatoses, a heterogeneous group of inherited skin disorders caused by pathogenic variants affecting skin structure and function. However, many genodermatoses remain molecularly uncharacterized, particularly in Middle Eastern populations. **Objectives:** This study aimed to evaluate the diagnostic accuracy and inherent challenges of utilizing (WES) for genodermatoses within a Middle Eastern context. **Methods:** We performed WES on three unrelated Middle Eastern pediatric patients presenting with genodermatoses. Genetic variants were prioritized and adjudicated according to ClinVar and the American College of Medical Genetics and Genomics (ACMG) guidelines. **Results:** WES identified pathogenic variants in three pediatric cases presenting with genodermatoses. Findings included a *GJB2* missense variant (c.148G>T; p.Asp50Asn) associated with keratitis–ichthyosis–deafness (KID) syndrome. This represents one of the first documented cases in a Middle Eastern population. Two additional patients presenting with epidermolysis bullosa harbored truncating variants in *COL7A1* (c.497dup; p.Val168Glyfs12) and *EXPH5* (c.5786del; p.Pro1929Leufs8), respectively; the latter also carried a *KRT5* missense variant (c.1607G>A; p.Ser536Asn). **Conclusions:** WES is a robust diagnostic adjunct for resolving ambiguity in rare genodermatoses, though its efficacy remains contingent on the availability of regional genomic references. Within pediatric dermatology, systematic exome sequencing serves as a powerful facilitator for transitioning from clinical suspicion to definitive molecular characterization. Collectively, these findings highlight the essential role of regionally representative genomic datasets in the accurate interpretation of novel variants and the advancement of precision dermatology.

## 1. Introduction

Genodermatoses are a heterogeneous group of inherited skin disorders caused by pathogenic variants in genes regulating skin structure and function. Comprising more than 500 distinct clinical entities, these disorders exhibit profound genetic diversity, with phenotypic expressions ranging from localized skin findings to life-threatening systemic pathologies involving the ocular, auditory, and musculoskeletal systems [1]. Although many of these phenotypes are clinically well characterized, their underlying genetic mechanisms remain incompletely understood, particularly in rare or atypical presentations [2]. Among the genodermatoses, Keratitis–Ichthyosis–Deafness (KID) syndrome (OMIM #148210) and epidermolysis bullosa (EB) serve as hallmark examples of how defects in intercellular communication and structural protein scaffolds lead to severe multisystem outcomes [3,4].

Keratitis-Ichthyosis-Deafness (KID) syndrome is an ultra-rare congenital ectodermal dysplasia with an estimated prevalence of less than 1 per 1,000,000 individuals worldwide (Orphanet ORPHA:477) and fewer than 100 cases documented in the global literature to date [5]. It is classically defined by a clinical triad of vascularizing keratitis, erythrokeratoderma, and sensorineural hearing loss, most commonly caused by pathogenic variants in *GJB2*, which encodes connexin 26. This protein is a critical component for maintaining ionic homeostasis and intercellular signaling within both the epidermis and the cochlear apparatus, explaining the systemic breadth of the syndrome [6].

In contrast, EB a clinically and genetically heterogeneous group of mechanobullous disorders characterized by marked skin fragility and blistering following minimal mechanical trauma [7]. Accurately ascertaining the global prevalence of epidermolysis bullosa remains challenging because geographic variations in consanguinity and the underreporting of milder phenotypes, particularly in underrepresented populations, likely result in a significant underestimation of the true disease burden [8]. Rather than a single disease entity, EB represents a spectrum of disorders defined by the level of skin cleavage within the dermal–epidermal junction and the underlying structural protein defect. Intraepidermal EB, encompassing EB simplex, results from disruption of the basal keratinocyte cytoskeleton and is most commonly associated with variants in *KRT5* and *KRT14*, encoding keratins 5 and 14, with additional causative genes including *PLEC*, *KLHL24*, *DST*, *EXPH5*, and *CD151*, which affect cytoskeletal organisation and cell–matrix stability. Junctional EB is characterised by cleavage within the lamina lucida and arises from variants in genes encoding hemidesmosomal and basement membrane components, including *LAMA3*, *LAMB3*, *LAMC2* (laminin-332), *COL17A1* (type XVII collagen), and integrin subunits *ITGA6* and *ITGB4*, with rarer involvement of *ITGA3*. Dystrophic EB involves dermal cleavage below the lamina densa and is caused by autosomal dominant or recessive variants in *COL7A1*, which encodes type VII collagen, the principal component of anchoring fibrils. Kindler EB represents a mixed-level blistering disorder caused by loss-of-function variants in *FERMT1*, encoding kindlin-1, a key regulator of integrin-mediated adhesion [9,10,11,12]. Collectively, these entities reflect a complex and sometimes overlapping genotype–phenotype spectrum across the structural hierarchy of the dermal–epidermal junction, a landscape that continues to expand as novel genetic drivers are identified. The increasing implementation of genomic technologies, particularly whole-exome sequencing (WES), has significantly enhanced the identification of disease-associated variants and expanded genotype–phenotype correlations within dermatology. However, diagnostic interpretation remains challenging in underrepresented populations, where a limited availability of population-specific genomic reference data may hinder accurate variant classification.

In this study, we utilized WES to investigate the genetic basis of rare genodermatoses in three unrelated patients of Middle Eastern origin. By identifying pathogenic variants associated with KID syndrome and EB, we aim to illustrate both the clinical utility and the inherent challenges of exome-based analysis in dermatology. Furthermore, we emphasize the importance of population-specific context in variant interpretation while highlighting the concordance of our findings with previously reported data in other cohorts.

## 2. Materials and Methods

### 2.1. Ethical Approval and Study Subjects

This study was conducted in accordance with the Declaration of Helsinki and was approved by Institutional Review Board (IRB) (Protocol Number: IRB: DER.MK.01) at American University of Beirut Medical Center (AUBMC). Recruitment of the families was conducted through the Genodermatoses Unit at the Department of Dermatology, AUBMC. The referring physician provided clinical phenotypic information. Written informed consent was obtained from all participants or their legal guardians. Three unrelated Lebanese families presenting with cutaneous or hair disorders were included in this study. Patient 1 was diagnosed with keratitis–ichthyosis–deafness (KID) syndrome and patients 2 and 3 were affected by epidermolysis bullosa.

Peripheral blood samples (5 mL) were obtained from participating patients and stored at 4 °C. Genomic DNA was extracted using the QIAamp Blood Midi Kit (QIAGEN Sciences, Inc., Germantown, MD, USA) in accordance with the manufacturer’s protocol. DNA concentration and purity were assessed using a NanoDrop spectrophotometer (Thermo Fisher Scientific, Inc., Waltham, MA, USA) at the Molecular Core Facility of the American University of Beirut. Subsequently, 5 µg of anonymized genomic DNA from each sample were shipped to Macrogen for whole-exome sequencing.

### 2.2. Whole-Exome Sequencing

Whole-exome sequencing (WES) and data analysis were performed as previously described [13]. Briefly, WES was conducted by Macrogen Inc., Seoul, Republic of Korea (https://dna.macrogen.com/) using the Illumina NovaSeq 6000 platform. Paired-end sequencing was performed with 100 bp reads following library preparation according to the manufacturer’s protocol. In brief, genomic DNA was randomly fragmented, followed by adapter ligation and library amplification using PCR. Cluster generation was performed on a flow cell via bridge amplification, and sequencing was carried out to produce raw image data. Base calling was performed using Illumina Real Time Analysis (RTA) software (Version 1.7.2), and raw BCL files were converted into FASTQ format using bcl2fastq for downstream analysis. Sequencing quality achieved an average Phred score of approximately Q33, corresponding to a base-calling accuracy of 99.9%. Additional sequencing quality metrics are summarized in Table 1.

### 2.3. Sequence Alignment, Variant Annotation, and Filtering Strategy

Raw sequencing data (FASTQ files) were aligned to the human reference genome (GRCh38/hg38) using CLC Genomics Workbench (version 20.0.4). Low-quality, failed, and fragmented reads were excluded from downstream analysis. Variant calling was performed using a minimum coverage threshold of 10×, requiring at least two supporting reads and a minimum variant allele frequency of 35%. The ploidy setting was defined as diploid (2), and genomic positions with excessively high coverage (>100,000 reads) were excluded to minimize artefactual alignment. For each sample, Binary Alignment Map (BAM) and Variant Call Format (VCF) files were generated containing all detected variants.

Variant annotation was conducted using Illumina Variant Studio (version 3.0), incorporating data from dbSNP, ClinVar, and the 1000 Genomes Project. A stringent filtering filter was then applied, retaining variants with a read depth > 20 and a minor allele frequency (MAF) of <1% based on population databases, including gnomAD [14]. Variants were limited to single nucleotide variants (SNVs), insertions, and deletions (indels), with functional prioritization given to predicted high-impact classes, including nonsense, missense, frameshift, and canonical splice-site variants.

To further refine candidate variants, each sample was compared against an in-house database of over 300 Lebanese exomes to exclude recurrent population-specific benign variants. Known disease-associated genes relevant to the phenotype were initially assessed; in their absence, the analysis focused on rare, potentially deleterious variants with high predicted pathogenicity. All identified variants were classified according to the American College of Medical Genetics and Genomics (ACMG) 2015 guidelines [15]. Variant interpretation was performed using VarSome as a supportive tool for evidence aggregation, including population frequency data, in silico predictions, and published literature. Final variant classification was determined by integrating all available evidence in accordance with ACMG criteria. Variants were also cross-referenced and linked to ClinVar where applicable for reported clinical significance. In silico functional impact was prioritized using Combined Annotation Dependent Depletion (CADD) scores. A scaled CADD score ≥ 20 was utilized as a threshold for deleteriousness, as this signifies that the variant is predicted to be among the top 1% of the most deleterious substitutions within the human genome [16]. Candidate variants were subsequently curated through a detailed literature review and interrogation of OMIM to confirm gene–disease associations and evaluate reported phenotypic relevance. Inheritance patterns were inferred based on family structure, zygosity, and segregation of the phenotype within the pedigree. Finally, BAM files were visualized using the Integrative Genomics Viewer (IGV; Broad Institute) to validate read alignment and confirm variant calls.

## 3. Results

Genetic analysis was completed for three unrelated index patients presenting with KID syndrome and EB. In total, five individuals were sequenced, including affected and unaffected family members where available. The identified variants included a known pathogenic variant associated with keratitis–ichthyosis–deafness syndrome and epidermolysis bullosa. The individual genetic findings for each patient are presented in the following sections.

### 3.1. Patient 1: GJB2 Variant Associated with KID Syndrome

**Figure 1:**
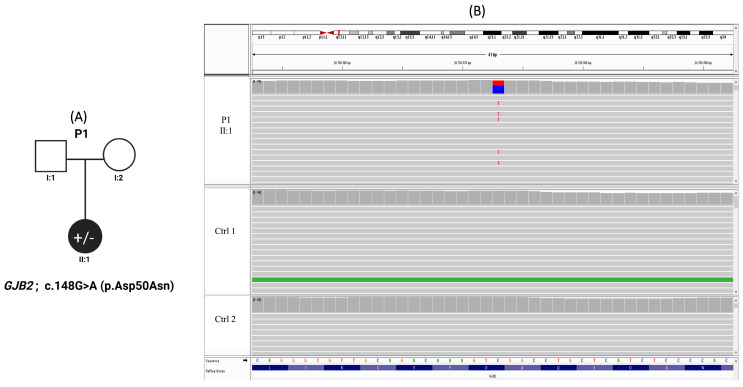
Pedigree and IGV confirmation of patient 1 of the *GJB2* variant in KID syndrome. (**A**) Pedigree of Patient 1 with keratitis–ichthyosis–deafness (KID) syndrome carrying a heterozygous *GJB2* variant (c.148G>A; p.Asp50Asn). The proband (II:1) is indicated by a filled symbol. (**B**) Integrative Genomics Viewer (IGV) visualization of whole-exome sequencing data demonstrating the heterozygous *GJB2* variant (c.148G>A; p.Asp50Asn) in the proband (upper panel). The reference and alternate alleles are highlighted in blue and red, respectively. Representative control samples lacking the variant are shown in the lower panels.

Congenital deafness accompanied by characteristic cutaneous manifestations prompted genetic evaluation in a 4-year-old female with a clinical diagnosis of KID syndrome. There was no reported family history of similar clinical features, and both parents were clinically unaffected and non-consanguineous; however, parental DNA samples were unavailable for genetic analysis (Figure 1A). Genetic analysis identified a heterozygous missense variant in *GJB2* (c.148G>A; p.Asp50Asn). Accordingly, the inheritance pattern is presumed to be either autosomal dominant or de novo.

This variant is rare in population databases and was absent from an internal cohort of 300 Lebanese exomes. It has been previously reported in individuals with KID syndrome, while remaining absent from large population reference datasets, further supporting its rarity in the general population. It is also classified as pathogenic in ClinVar. It was detected with a read depth of 104, supporting high confidence in variant calling. In silico prediction supports a deleterious effect on protein function, with a CADD score of 23.7. Based on ACMG classification, the variant is interpreted as pathogenic, supported by multiple lines of evidence including computational predictions, prior disease association, and population data (Table 2). Variant confirmation was performed using Integrative Genomics Viewer (Figure 1B).

### 3.2. Patient 2: COL7A1 Variant Associated with Epidermolysis Bullosa

**Figure 2:**
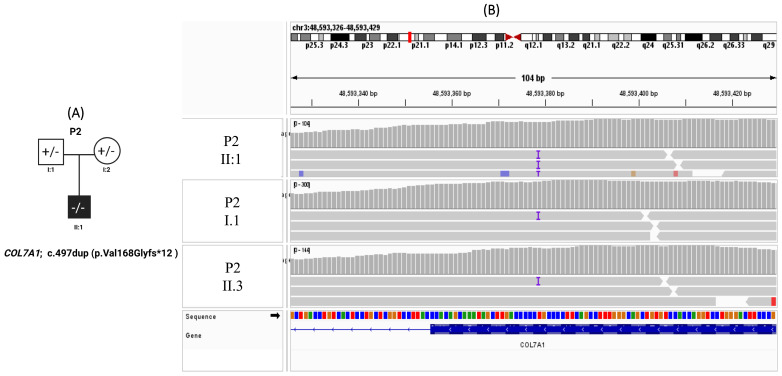
Pedigree and IGV confirmation of patient 2 of the *COL7A1* variant in epidermolysis bullosa. (**A**) Pedigree of Patient 2 with epidermolysis bullosa carrying a frameshift variant in COL7A1 (c.497dup; p.Val168Glyfs*12). The proband (II:1) is indicated by a filled symbol. Parental carrier status is shown as heterozygous. (**B**) Integrative Genomics Viewer (IGV) visualization of whole-exome sequencing data demonstrating the COL7A1 variant in the proband (P2 II:1, upper panel) and segregation in family members. The variant is present in heterozygous form in both parents and homozygous for the proband.

Patient 2 is a male infant who was born with a large bullous lesion over the back and persistent vomiting shortly after birth. Given the clinical suspicion of epidermolysis bullosa with pyloric atresia, diagnostic imaging was performed and confirmed the presence of pyloric atresia as the cause of the vomiting due to anatomical obstruction. Surgical correction was subsequently undertaken (Figure 2A). At 18 months of age, the patient remains alive but demonstrates failure to thrive, recurrent upper respiratory tract infections, and generalized bullae involving both the skin and mucosal surfaces. WES was performed, with initial analysis focusing on known epidermolysis bullosa–associated genes, including *ITGA6*, *ITGB4*, and *PLEC*, none of which revealed pathogenic variants. Further analysis identified a homozygous frameshift variant in *COL7A1* (c.497dup; p.Val168Glyfs*12) (Figure 2B), with a sequencing depth of 65×. The variant was absent from an internal cohort of 300 Lebanese exomes and is extremely rare in public databases. This variant is predicted to result in premature protein truncation and was classified as pathogenic according to ACMG criteria and ClinVar. (Table 2). Segregation analysis established an autosomal recessive inheritance pattern, confirming the patient as homozygous for the variant. Both parents were identified as asymptomatic heterozygous carriers; notably, there was no reported consanguinity in the family history.

### 3.3. Patient 3: Severe Epidermolysis Bullosa with EXPH5 Pathogenic Variant and a Possible Modifier KRT5 Variant

**Figure 3:**
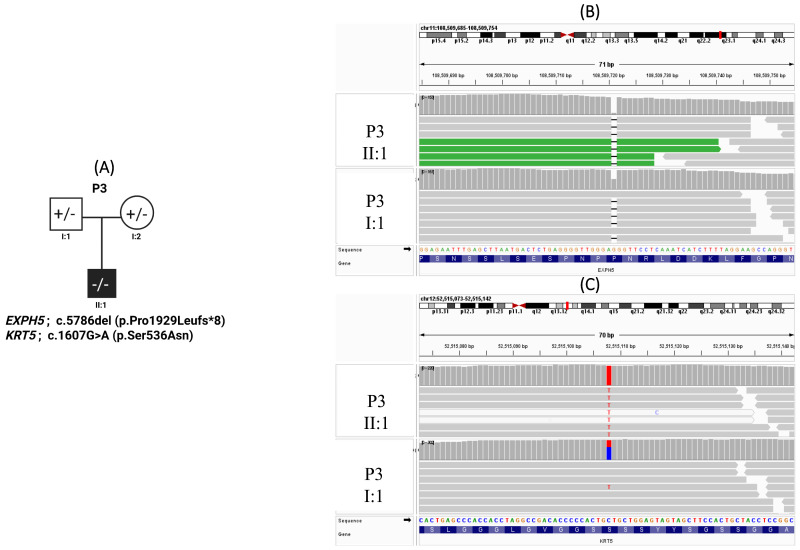
Pedigree of Patient 3 with epidermolysis bullosa carrying *EXPH5* and *KRT5* variants. (**A**) Pedigree of Patient 3 with epidermolysis bullosa harboring a frameshift variant in *EXPH5* (c.5786del; p.Pro1929Leufs*8) and a missense variant in *KRT5* (c.1607G>A; p.Ser536Asn). The proband (II:1) is indicated by a filled symbol. (**B**) Integrative Genomics Viewer (IGV) visualization of the *EXPH5* variant showing homozygosity in the proband (upper panel) and heterozygous carrier status in I:1 (lower panel). (**C**) IGV visualization of the *KRT5* variant demonstrating homozygosity in the proband (upper panel) and heterozygosity in individual I:1 (lower panel). The reference and alternate alleles are highlighted in blue and red, respectively.

Patient 3 is a male neonate who presented shortly after birth with diffuse cutaneous and mucosal bullae and erosions. Additional findings included nail involvement characterized by granulation tissue and bleeding, hoarseness of voice, and failure to thrive. The clinical course was complicated by recurrent infections. Despite supportive care, the patient developed septic shock and died at the age of two months. (Figure 3A).

WES identified a homozygous frameshift variant in *EXPH5* (c.5786del; p.Pro1929Leufs*8), predicted to result in loss of normal protein function and (Figure 3B). The variant was absent from an internal cohort of 300 Lebanese exomes and is extremely rare in public population databases. It was detected at a read depth of 93× and classified as pathogenic according to ACMG criteria and ClinVar. While maternal DNA was unavailable, the patient’s homozygosity and the father’s heterozygous status strongly support an autosomal recessive model, with the mother presumed to be a heterozygous carrier. Both parents were clinically unaffected and non-consanguineous. In addition, a homozygous missense variant in *KRT5* (c.1607G>A; p.Ser536Asn) was identified, with a read depth of 150× and a CADD score of 20.3 (Figure 3C, Table 2). It was absent from our in-house exome cohort and classified as a variant of uncertain significance (VUS) according to ACMG criteria and ClinVar (Figure 3C, Table 2).

## 4. Discussion

In this study, WES was utilized to investigate the molecular basis of rare genodermatoses in three unrelated patients. These findings illustrate the role of genomic integration in refining clinical diagnoses, particularly in underrepresented populations where interpreting rare variants remains a significant challenge.

### 4.1. Population-Specific Confirmation of a Pathogenic GJB2 Variant in KID Syndrome

GJB2 encodes connexin 26, a key gap junction protein involved in intercellular communication in epithelial tissues and the inner ear, with an established role in cochlear homeostasis and epidermal integrity [17]. The *GJB2* c.148G>A (p.Asp50Asn) variant has been reported sporadically worldwide. Asp50 is located within the first extracellular loop of connexin 26, a region critical for connexon–connexon docking and channel permeability, and substitution of a negatively charged aspartic acid with a neutral asparagine is predicted to alter intercellular channel function [6]. Within the Middle Eastern North African (MENA) region, it has been documented only once, in an Egyptian individual, where it was identified as de novo [14]. Interrogation of population-level sequencing resources, including the Catalogue for Transmission Genetics in Arabs (CTGA), confirms the absence of this variant across Middle Eastern population. Outside the MENA region, this variant has been reported in isolated, genetically confirmed cases from Iran, Argentina, and sub-Saharan Africa [18,19,20].

Here, we report the first molecularly confirmed case of *GJB2* c.148G>A (p.Asp50Asn) in the Middle East, further expanding the geographic and molecular characterization of this rare variant. In the absence of parental genetic testing, the inheritance pattern could not be definitively established. However, the lack of reported family history in both parents suggests either a de novo pathogenic variant or autosomal dominant inheritance with incomplete penetrance. This interpretation is supported by the well-documented variability in clinical expressivity and incomplete penetrance associated with *GJB2*-related disorders, whereby pathogenic variants in connexin 26 may be present in individuals with absent or very mild clinical manifestations, as demonstrated in previous genotype–phenotype studies [21,22,23]. The inclusion of *GJB2* c.148G>A (p.Asp50Asn) in Middle Eastern molecular datasets is important for several reasons. First, genetic reference resources from the Middle East remain underrepresented relative to other populations, and the absence of region-specific data can lead to uncertainty in variant interpretation. From diagnostic perspective, documenting this variant in a Middle Eastern context contributes to underrepresented regional genomic data and supports its inclusion in comprehensive hearing loss and syndromic ectodermal disorder gene panels. Incorporation of rare, well-characterized variants such as c.148G>A enhances diagnostic yield, facilitates early molecular confirmation in prenatal or early-life testing settings, and informs genetic counseling, particularly in populations where genomic reference data remain limited.

### 4.2. Structural Disruption in Dystrophic Epidermolysis Bullosa: COL7A1 Frameshift Variant

In Patient 2, clinical and molecular findings were consistent with dystrophic epidermolysis bullosa (DEB), in which a frameshift variant was identified in *COL7A1*, the gene encoding type VII collagen. Type VII collagen is the principal structural component of anchoring fibrils, which are essential for maintaining dermal–epidermal adhesion at the basement membrane zone [24]. Disruption of anchoring fibril assembly results in mechanical fragility below the lamina densa, giving rise to the characteristic dermolytic blistering observed in DEB. The identified frameshift variant is predicted to result in loss of functional type VII collagen, consistent with a loss-of-function mechanism typically associated with autosomal recessive dystrophic epidermolysis bullosa (RDEB) [25,26]. In contrast to dominant-negative glycine substitution variants observed in autosomal dominant DEB, truncating variants such as frameshift variants impair anchoring fibril formation and are generally associated with more severe clinical phenotypes. Although segregation analysis was not available to confirm inheritance, the molecular characteristics of the variant, together with the clinical presentation, support classification within the recessive DEB spectrum. Furthermore, this variant has been reported in multiple unrelated individuals with DEB, supporting its pathogenic relevance and recurrence in disease cohorts [9,24,26,27,28,29].

This patient also presented with pyloric atresia, a feature classically associated with epidermolysis bullosa with pyloric atresia (EB-PA), which is most commonly linked to pathogenic variants in *ITGA6* and *ITGB4*, and less frequently *ITGA3*, within the junctional EB spectrum. Gastrointestinal obstruction in the form of pyloric atresia is not a recognised defining feature of *COL7A1*-associated DEB, and this combination therefore represents an atypical genotype–phenotype association.

However, extracutaneous involvement in *COL7A1*-related disease has been documented. Previous reports have described esophageal stenosis and mucosal fragility in patients with recessive dystrophic EB, highlighting the role of type VII collagen in maintaining epithelial integrity beyond the skin [30,31,32]. Notably, such manifestations may occur even in the absence of significant cutaneous blistering, underscoring the variable expressivity of COL7A1-associated disease [31]. While pyloric atresia represents a distinct gastrointestinal phenotype, these findings suggest that anchoring fibril dysfunction may extend more broadly across gastrointestinal epithelia than previously recognized, a concept that warrants further investigation.

Taken together, this case supports the hypothesis that *COL7A1*-related disease may, in rare instances, manifest with an expanded gastrointestinal phenotype beyond classical descriptions. This finding suggests that type VII collagen may contribute more broadly to epithelial stability across multiple tissue types and may reflect shared structural vulnerabilities and convergent pathogenic mechanisms within the basement membrane zone. It may also indicate the presence of additional genetic or developmental modifiers that influence phenotypic expression beyond the primary pathogenic variant. Future studies integrating deep phenotyping with comprehensive genomic analyses will be essential to test this hypothesis and clarify the underlying mechanisms. Improved resolution of genotype–phenotype correlations in epidermolysis bullosa will be critical for enhancing diagnostic precision and defining the full systemic spectrum of *COL7A1*-associated disease.

### 4.3. Severe Epidermolysis Bullosa Due to EXPH5 Pathogenic Variant with Potential KRT5 Modifier Effect

Patient 3 presented with a severe, ultimately fatal epidermolysis bullosa (EB) phenotype and was found to carry homozygous variants in both *EXPH5* (c.5786del; p.Pro1929Leufs*8) and *KRT5* (c.1607G>A; p.Ser536Asn), suggesting a potentially complex genetic basis for disease severity.

The *EXPH5* variant is a frameshift predicted to result in premature truncation and loss of function, consistent with the established pathogenic mechanism underlying autosomal recessive epidermolysis bullosa simplex (EBS4), and is supported by ClinVar classification as well as prior reports of identical or functionally similar variants in affected individuals [33,34,35]. *EXPH5* encodes exophilin-5, a Rab27B effector involved in vesicle trafficking and actin cytoskeletal organization in keratinocytes [35]. Although biallelic loss-of-function variants in *EXPH5* are typically associated with mild to intermediate EB phenotypes and neonatal lethality has not been commonly reported, the severity observed in this patient substantially exceeds the expected disease spectrum, indicating that additional factors are likely contributing to phenotypic expression.

To explore this discrepancy, we hypothesized that additional genetic contributors may underlie the observed severity, prompting further variant analysis. This identified a homozygous missense variant in *KRT5* (p.Ser536Asn), classified as a variant of uncertain significance in ClinVar, supported by very low population frequency, and with in silico evidence of potential functional impact (CADD score 20.3). *KRT5* encodes keratin 5, a key structural protein of basal keratinocytes that forms intermediate filaments with keratin 14 and provides mechanical stability to the epidermis [36]. While most KRT5-associated epidermolysis bullosa simplex is autosomal dominant, emerging evidence demonstrates that biallelic loss or severe impairment of KRT5 can result in autosomal recessive EB, including very severe and even lethal phenotypes, reflecting the essential role of keratin 5 in cytoskeletal integrity [37,38,39]. Therefore, this case raises the possibility that the *KRT5* variant could modify disease severity in the setting of *EXPH5* deficiency. In this context, the *KRT5* variant may act as a disease modifier in the setting of *EXPH5* deficiency, a concept supported by growing evidence for genetic modifiers in EB affecting interconnected structural pathways [40,41]. However, this remains hypothetical and requires confirmation through segregation studies and functional validation. Alternative explanations include an extreme presentation of *EXPH5*-related disease or additional undetected genetic or environmental contributors. Overall, this case suggests a severe EB phenotype driven by pathogenic *EXPH5* loss of function, with a likely modifying contribution from homozygous *KRT5*, potentially exacerbating cytoskeletal fragility and disease severity.

Overall, these findings support the role of pathogenic variants in established genodermatosis genes as primary disease drivers, with potential contribution from additional modifying variants in selected cases. However, interpretation of complex or expanded phenotypes remains limited in the absence of functional studies and segregation data.

## 5. Conclusions

This study highlights the clinical utility of WES in resolving the molecular basis of different types of genodermatoses, including EB and KID syndrome. WES enabled the identification of pathogenic variants in established disease-associated genes, confirming clinical diagnoses and providing population-specific insights from the Middle East. These findings reinforce the value of WES as a first-line diagnostic tool in well-characterized Mendelian disorders with overlapping phenotypic presentations. At the same time, this study underscores the limitations of exome-based analysis in resolving complex or expanded phenotypes. In the absence of segregation studies and functional validation, interpretation of rare variants and potential modifying effects remains constrained. Future integration of comprehensive genomic approaches, including copy number variant detection, alongside functional studies, will be essential to improve diagnostic precision and further refine genotype–phenotype correlations in genodermatoses.

## Figures and Tables

**Table 1:** Summary of sequencing quality metrics for patients and family members.

Sample	Read Count	GC (%)	AT (%)	Q20 (%)	Q30 (%)
Patient 1	42,879,658	52.17	47.83	97.54	93.55
Patient 2	28,687,479	52.1	47.9	97.95	95.1
Father of Patient 2	41,385,272	52.54	47.46	97.83	94.96
Mother of Patient 2	31,251,167	52.29	47.71	97.91	94.94
Patient 3	30,577,148	52.43	47.57	97.72	94.98
Father of Patient 3	34,126,712	52.32	47.68	97.86	95.11

**Table 2:** Genetic and Clinical Characteristics of the Patients.

Genetic Findings								Clinical Findings			
Patient	Gene Variants; (Read Depth)	Coding Impact	CADD * Score	ACMG * Classification	Predicted Effect (ClinVar)	MAF * (GnomAD v2.1.1)	MAF (Lebanese Exomes)	Primary Clinical Phenotype	Hair/Nail/Mucosal Involvement	Associated Features	Presumed Inheritance
Patient 1	*GJB2* (c.148G>A; p.Asp50Asn); (104)	Missense	23.7	Pathogenic—PS3 (S)|PS2(S)|PM1 (M)|PP2 (SP)|PM2 (M)|PM5 (M)|PP4 (VS)	Pathogenic	0	0	KID * syndrome	Ichthyosis, alopecia	Congenital deafness	De novo/Autosomal Dominant
Patient 2	*COL7A1* (c.497dup; p.Val168Glyf*12); (65)	Frameshift	NA	Pathogenic—PS4 (M)|PVS1 (VS)|PM2(M)|PS3 (S)	Pathogenic	4.446 × 10^−5^	0	Epidermolysis bullosa with pyloric atresia	NA	Pyloric atresia, * URTI, * FTT	Autosomal recessive
Patient 3	*EXPH5* (c.5786del; p.Pro1929Leufs*8); (93)	Frameshift	NA	Pathogenic-PM3(S)|PS3 (S)|PM2 (M)|PVS1 (M)	Pathogenic	0.000024	0	Epidermolysis bullosa	Mucosal bullae and erosions, nail involvement with granulation tissue and bleeding.	Sore voice, * FTT.	Autosomal recessive
	*KRT5* (c.1607G>A; p.Ser536Asn); (150)	Missense	20.3	VUS-PM3 (M)|PM2 (SP)|PP4 (SP)|PP2 (SP)	Uncertain Significance	0.000087	0				

* CADD: Combined Annotation Dependent Depletion. ACMG: American College of Medical Genetics and Genomics. VUS: variant of uncertain significance. MAF: minor allele frequency. KID: keratitis–ichthyosis–deafness syndrome. FTT: failure to thrive. URTI: upper respiratory tract infections. ACMG classification was carried out using VarSome (VS—Very Strong; S—Strong; M—Moderate; SP—Supporting).

## Data Availability

The original contributions presented in this study are included in the article. Further inquiries can be directed to the corresponding authors.
